# Adherence to NICE Guidelines and Centor Criteria in Acute Sore Throat Management: An Audit Cycle

**DOI:** 10.7759/cureus.92540

**Published:** 2025-09-17

**Authors:** Cameron Lynch

**Affiliations:** 1 Otolaryngology, Fairfield General Hospital, Manchester, GBR

**Keywords:** accident and emergency, antibiotic prescribing, centor score, clincal audit, quality improvement, sore throat

## Abstract

Introduction

Antibiotic stewardship is becoming critically important as resistant bacteria become increasingly prevalent. Antimicrobial resistance (AMR) has emerged as a major global health challenge, driven in part by the misuse and overuse of antibiotics. An acute sore throat is one of the most frequent reasons for patients to seek medical care, yet only a minority of cases require antibiotic treatment. Despite this, inappropriate prescribing is rife, often influenced by diagnostic uncertainty, patient expectations, and non-clinical pressures. These challenges highlight the importance of reinforcing evidence-based strategies, such as the use of Centor criteria, to support accurate assessment and reduce unnecessary antibiotic prescribing in patients presenting with a sore throat.

This quality improvement project evaluated adherence to Centor criteria and subsequent antibiotic prescribing in an accident and emergency (A&E) department in the Northwest of England, before and after clinician education on NICE guidance and documentation of Centor criteria.

Materials and methods

One hundred patients presenting with an acute sore throat between January and March 2023 were identified. Electronic patient data were collected retrospectively to analyze the documentation’s adherence to NICE guidelines and Modified Centor criteria.

Results

Pre-intervention, 16% of clinicians documented all Centor criteria, 48% incorrectly prescribed antibiotics, and only 20% prescribed appropriate antibiotics. Through post-intervention teaching and literature dissemination, the figures changed to 54%, 22%, and 56%, respectively.

Conclusion

Targeted clinician education and adherence to NICE guidelines improved antibiotic prescribing for the acute sore throat, underscoring the value of audit cycles while highlighting the need for sustained, larger-scale efforts to reduce AMR.

## Introduction

Antibiotic stewardship is becoming increasingly important as antimicrobial resistance (AMR) continues to grow. The World Health Organization attributes the rise in AMR primarily to the misuse and overuse of antimicrobials. The consequences of escalating resistance include higher morbidity and mortality, prolonged infections, longer hospital stays, increased reliance on expensive second-line treatments, and a greater likelihood of antibiotic therapy failure [1,2].

An acute sore throat is a common presentation to accident and emergency (A&E) and primary care. NICE estimates its incidence in the United Kingdom (UK) general practice at 100 per 1,000 population annually, with 38% of adults attending primary care or A&E at some point in their lives for this reason [3,4]. A study of 600 patients with acute tonsillitis found that only 24% genuinely required antibiotics, highlighting ongoing inappropriate prescribing [5]. Additionally, an analysis of prescribing trends from 2014 to 2022 revealed that the Northwest of England, characterized by higher deprivation, accounted for a disproportionate number of antibiotic prescriptions compared to other regions in the UK [6].

Although bacteria contribute to only 5-30% of acute sore throat cases, differentiating between viral and bacterial causes remains challenging due to overlapping clinical features [7,8]. A large meta-analysis concluded that a Centor score of 0 effectively excludes bacterial infection, a point reiterated in NICE guidelines [9,10]. NICE guidelines recommend either the Centor or FeverPAIN criteria for diagnosing sore throat, and the Centor scoring was chosen due to its practicality and stronger evidence base, particularly in non-primary care settings. Compared with FeverPAIN, Centor provides a more robust and standardizable tool, supported by meta-analysis evidence demonstrating its reliability in ruling out bacterial infection [9]. Public Health England also highlighted the ongoing issue of antibiotic overprescription, noting that 59% of patients presenting with a sore throat received antibiotics, despite expert opinion suggesting that only around 11% actually required them [11].

Outside the scope of acute sore throat, studies indicate that inappropriate antibiotic use is higher among patients with multiple comorbidities, often linked to socio-economic factors, where clinicians worry about potential complications [12]. However, some literature suggests that socio-economic status may be less influential in prescribing decisions for upper respiratory tract infections [13].

Patient and parental expectations heavily influence prescribing patterns, especially in pediatric care. One study examining antibiotic use for sore throat in A&E found that 29.1% of prescriptions were inappropriate, often driven by fears of missing severe illness, patient pressure, and misconceptions about antibiotics, factors exacerbated by gaps in clinician and patient education [14].

Interestingly, some clinicians admitted to prescribing antibiotics inappropriately, fully aware of their actions, citing time pressures, anxiety about the potential for rapid deterioration in pediatric cases, and the impact of COVID-19 as contributing factors [15]. Similar findings have been reported in a systematic review, which identified non-clinical factors such as department waiting times and perceived patient ideas, concerns, and expectations as significant influences on prescribing behavior [16]. These factors underscore the critical need for improved education for both patients and clinicians regarding the appropriate use of antibiotics in cases of acute sore throat.

This two-cycle audit focused on an A&E department in the Northwest of England. The project’s aim was to assess adherence to the use of Centor criteria in clinician documentation for sore throat. The recorded documentation was used to assess the appropriateness of clinicians’ antibiotic prescribing, including whether a prescription was necessary, the correct antibiotic was selected, and whether delayed dispensing was appropriately applied. The goal was to implement judicious antibiotic use in the assessment and management of sore throat presentations.

## Materials and methods

Patients presenting to a single A&E department of a district general hospital in the Northwest of England, triaged with an acute sore throat between January and March 2023, were identified via the local trust data collection department and electronic patient records (EPRs). Relevant triage phrases included “acute sore throat,” “throat pain,” “neck pain,” “odynophagia,” “pharyngitis,” “tonsillitis,” “painful swallow,” “burning throat,” “trismus,” and “laryngitis.” Inclusion criteria encompassed all adult and pediatric patients with these triage presentations, while cases later attributed to alternative causes (e.g., trauma-related neck pain or foreign-body-induced dysphagia) were excluded. Ethnicity was not recorded.

The study assessed adherence to Centor criteria documentation, appropriateness of antibiotic prescribing, correctness of antibiotic choice, correct non-prescribing decisions, and inappropriate prescribing. Targets were 100% adherence for all outcomes except inappropriate prescriptions, where the target was 0%.

During the first audit cycle, 50 patients were analyzed retrospectively. Accessing the EPR for each patient, clinician documentation was reviewed for inclusion of Modified Centor criteria (Table 1) and adherence to NICE guidelines, which recommend supportive care for scores 0-2 and immediate or delayed antibiotics for scores ≥3.

**Table 1:** Modified Centor criteria (McIsaac) Adapted from S. Winslow, Taming the SRU. Reference [17,18].

Modified Centor criteria (McIsaac)	Score
Fever	1
Tonsillar exudate	1
Absent cough	1
Anterior cervical lymphadenopathy	1
Age: 3-14 years	1
Age: 15-44 years	0
Age: >44 years	-1

**Figure 1:**
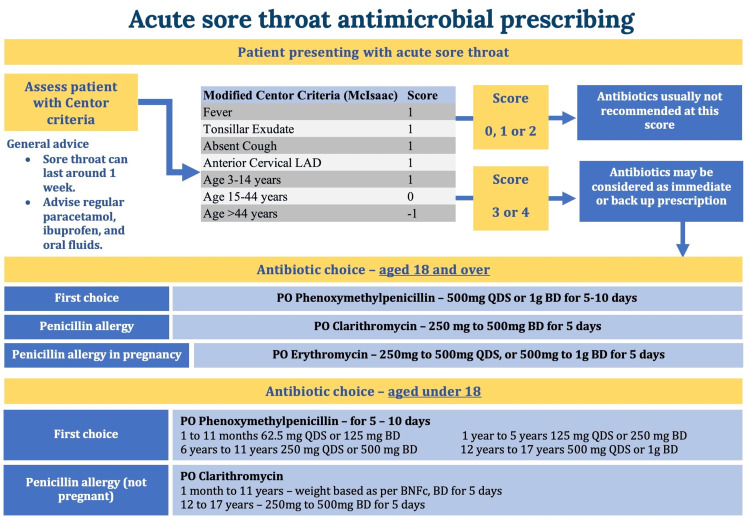
Education poster depicting the pathway of clinician assessment and management for acute sore throat, detailing the Modified Centor criteria Adapted from NICE Guideline NG84. LAD, lymphadenopathy; PO, oral medication; BD, twice daily; QDS, four times per day; BNFc, British National Formulary for Children Reference [10].

The NICE guideline 84 regarding the antibiotic choice for pediatric and adult patients presenting with an acute sore throat is displayed in the poster intervention in Figure 1 [10].

The intervention, implemented from February 2023, included clinician education and guideline dissemination. Three teaching sessions were delivered to junior doctors, advanced nurse practitioners, registrars, and consultants. A poster summarizing NICE guideline 84 (Figure 1) was displayed across A&E minors and majors, and the urgent treatment center. Teaching emphasized the correct use of the Centor criteria. Daily reminders at clinician handover ensured continuity of intervention implementation. Post-intervention, a second cohort of 50 patients was analyzed in March 2023 to evaluate the impact of the intervention.

## Results

Pre-intervention

A cohort of 50 patients was collected during the first cycle between January and February 2023 after fulfilling the inclusion criteria. The median age was 26 years (range, six months to 52 years). Only eight patients (16%) had complete Centor documentation, with cervical lymphadenopathy most frequently omitted.

To assess whether appropriate antibiotic choice had been prescribed in those patients lacking a formal score, a best effort was made from the documentation to infer a Centor criteria score and thus understand the clinician’s prescribing decisions. Fifteen patients (30%) scoring equal to or greater than 3 were appropriately prescribed antibiotics. Of those 15 patients, ten (20%) received the correct choice of antibiotics as per NICE. Eleven patients (22%) with scores ≤2 were appropriately not prescribed antibiotics, yet almost half (24, 48%) were inappropriately given antibiotics despite scoring <3.

Intervention

**Figure 2:**
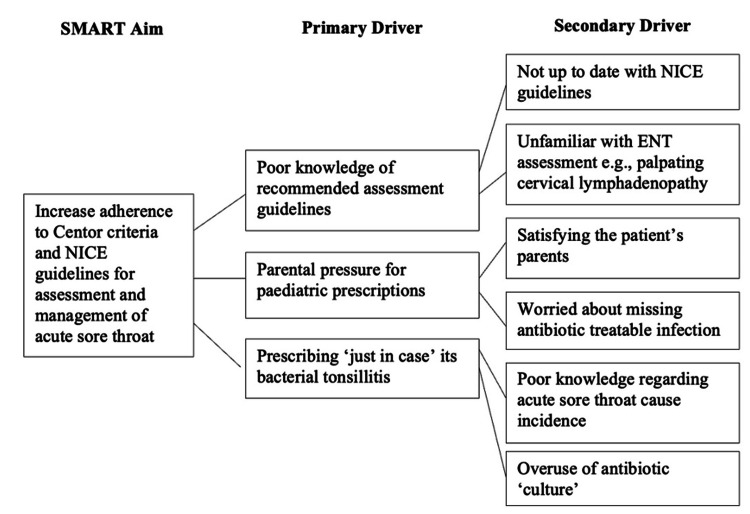
Driver diagram depicting the SMART aim with contributing primary and secondary drivers ENT, ear, nose, and throat; SMART, specific, measurable, achievable, relevant, and time-bound

Pre-intervention results highlighted significant gaps in the assessment and management of acute sore throat. To address these, a specific, measurable, achievable, relevant, and time-bound (SMART) aim (Figure 2) was set to improve adherence to Centor documentation and NICE prescribing guidelines. Staff and patient feedback identified primary and secondary drivers, including parental pressure in pediatric cases, a culture of “just in case” prescribing, and limited awareness of guidelines among A&E staff. A target of 20% improvement in Centor documentation and correct antibiotic use was set. Interventions included face-to-face clinician teaching, email dissemination of guidance, and poster displays (Figure 1), reinforced at daily handovers. Implemented over four weeks, these measures aimed to embed sustained changes in local prescribing practice. 

Post-intervention 

Following the intervention, another cohort of 50 patients was retrospectively collected between late February and the end of March 2023. The median age was 29 years (range, 17 months to 62 years). Complete Centor documentation rose to 27 patients (16-54%). Appropriate prescribing for scores ≥3 improved to 28 patients (56%), with correct antibiotic choice in 24 of those 28 patients (86%). Appropriate non-prescribing for scores ≤2 remained stable at 11 patients (22%). Most notably, inappropriate prescriptions for scores <3 halved, falling from 48% (24 patients) to 22% (11 patients).

**Figure 3:**
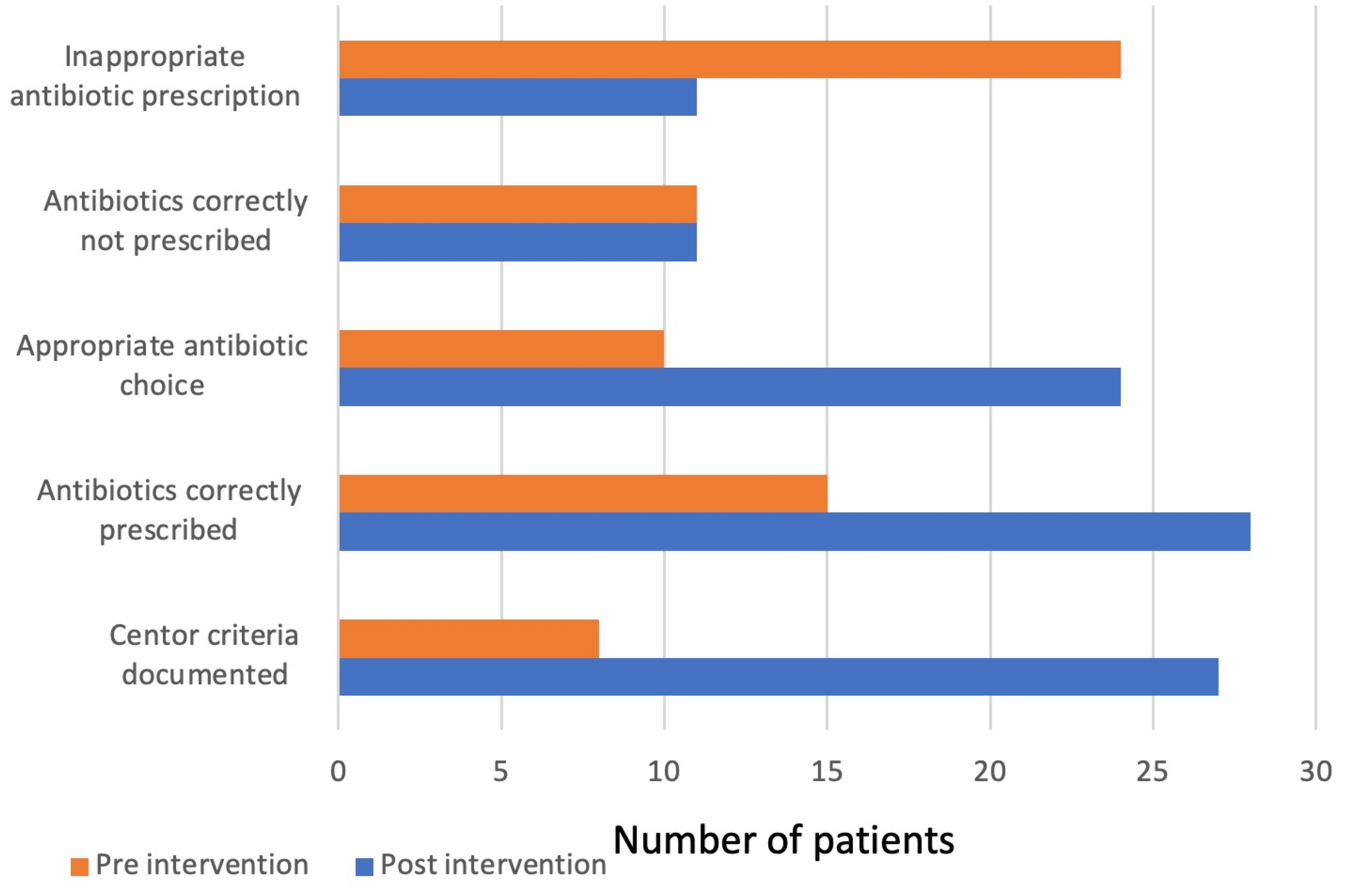
Bar graph demonstrating the difference in patient numbers pre- and post-intervention for the identified targets.

The success of the interventions is graphically displayed in Figure 3 and as targeted percentages in Table 2. 

**Table 2:** Improvements noted in the aforementioned targeted criteria following intervention implementation

Standard	Target	Cycle 1	Cycle 2
Centor criteria documentation	100%	16%	54%
Appropriately prescribed antibiotics	100%	30%	56%
Of those prescribed antibiotics, the correct antibiotic choice	100%	75%	85.70%
Correctly not prescribed antibiotics	100%	22%	22%
Inappropriately prescribed antibiotics	0%	48%	22%

## Discussion

An acute sore throat is a common presentation in both primary and secondary care in the UK. The burden of inappropriate antibiotic prescribing and its contribution to AMR are well recognized. Addressing this requires judicious prescribing, with individual clinicians carrying the responsibility to drive change. This audit cycle highlighted suboptimal adherence to NICE prescribing guidelines at a local level. Following targeted intervention, measurable improvements were achieved.

Our intervention comprised staff education delivered through teaching sessions and posters, with reinforcement achieved through repetition of key messages and continuity at clinician handover. As a result, adherence improved in four of five audit targets: documentation of Centor criteria, correct prescribing, and appropriate antibiotic choice each rose by over 26%, while unnecessary prescriptions were reduced by more than half.

These findings reflect wider evidence on inappropriate prescribing. Agarwal et al. reported on a cohort of 600 patients with acute tonsillitis, where only 24% required antibiotics, yet prescribing exceeded this substantially [5]. Despite its single-center design and lack of adjustment for clinician variability, the study’s sample size highlights a consistent problem across healthcare systems. Similarly, Public Health England found that 59% of sore throat patients received antibiotics, although expert consensus estimated only 11% required them [11]. Although descriptive and unadjusted for confounders such as comorbidities or patient expectations, the dataset’s national scale adds weight to concerns of overprescribing. Together, these studies contextualize our audit by demonstrating that local practice mirrors national and international trends.

Regarding the diagnostic approach, NICE guidelines recommend either the Centor or FeverPAIN criteria [10]. We focused on Centor for practical and methodological reasons; unlike FeverPAIN, which includes more subjective features such as “rapid onset” and “severely inflamed tonsils,” Centor is less vulnerable to inter-clinician interpretation and variation. A meta-analysis by Willis et al. supports this, showing that Centor at lower thresholds offers stronger negative predictive value for ruling out bacterial infection [9]. While heterogeneity between studies was a limitation, the pooled dataset provides higher-level evidence than individual cohorts. Thus, Centor was the most robust and standardizable tool for our audit setting.

Regional variation also warrants attention. McCloskey et al. analyzed prescribing between 2014 and 2022 and identified disproportionately high antibiotic use in the Northwest of England, particularly in deprived areas [6]. Although causality cannot be inferred from its ecological design, the longitudinal nature and large dataset strengthen the conclusion that socio-economic factors and local culture influence prescribing. Our audit, conducted in this region, provides a micro-level perspective that reinforces these findings.

Finally, drivers of inappropriate prescribing extend beyond clinical uncertainty. Ahmad et al. found that 29.1% of emergency department prescriptions for sore throat were inappropriate, largely influenced by patient expectations and clinician anxiety [14]. Hampton et al. similarly showed, through qualitative interviews, that some clinicians knowingly prescribed inappropriately due to time pressure, fear of pediatric deterioration, and pandemic-related uncertainty [15]. Although qualitative studies lack statistical power, they add valuable insight into the pressures shaping prescribing behavior. Lim et al.’s systematic review further synthesized these influences, identifying non-clinical factors such as waiting times and perceived patient concerns as consistent drivers across emergency settings [16]. Collectively, this evidence supports our intervention strategy, educating clinicians to address both knowledge gaps and behavioral determinants.

This audit had several strengths, including rapid data collection, timely implementation of multiple interventions, and clear improvements in prescribing practice and Centor documentation. The challenge moving forward is to sustain these behaviors, particularly among new clinicians, and to share these lessons across the trust. Extending this approach to other conditions where prudent antibiotic use is essential could have broader benefits. Ultimately, a trust-wide reaudit with a larger dataset would provide a more representative picture of regional prescribing habits and contribute to wider AMR reduction efforts.

Despite the large sample size and broad age inclusion, several limitations must be acknowledged. Selection bias may have occurred at the triage stage, as some patients were missed due to inappropriate triage, complex presentations, or leaving prior to clinician review. Observer bias and study design warrant consideration, both in clinical assessments and in the interpretation of documentation, which may not have fully reflected clinical reasoning. Similarly, over-reliance on Centor scoring may have influenced prescribing decisions; however, clinicians should retain the ability to override scoring systems when clinical judgment indicates otherwise. The single-center nature of the audit and relatively small sample size limit generalizability. Additionally, most acute sore throat presentations were managed in the Urgent Treatment Centre, often by a small group of non-doctor clinicians, which may have introduced bias. The lack of recorded streptococcal testing, particularly in pediatric patients, could have implications for antibiotic prescribing decisions independent of Centor score performance. 

Despite a correlation being observed regarding a higher scoring Centor score and need for antibiotics, a causal relationship cannot be established. Repeating further audit cycles could assess the effect of confounding variables such as seasonal symptom variation, Hawthorne effect, and rotation of clinical staff, in addition to implementing across multiple trusts with longer patient follow-up. Another limitation encountered during data collection was incomplete documentation of the Centor criteria. At times, it was necessary to infer clinicians’ rationale for antibiotic prescribing from EPR entries, which may have introduced confirmation bias during retrospective analysis. Further statistical analysis of the recorded data could strengthen the conclusions and potentially reveal significance in the interventions. However, post-intervention improvements were evident across all areas of A&E, highlighting the effectiveness and adaptability of the interventions.

## Conclusions

This audit demonstrates that targeted interventions, such as clinician education on Centor criteria and adherence to NICE guidelines, can meaningfully improve antibiotic prescribing for acute sore throat. The findings have implications beyond the local setting, highlighting how structured audit cycles can inform wider clinical practice. Sustained efforts and larger-scale audits will be essential to maintain improvements and contribute to the broader effort to reduce AMR.
